# Good neighbours: current-year needles in Nordmann fir rely on their 1-year-old neighbouring needles for adequate nutrient supply

**DOI:** 10.1093/treephys/tpag064

**Published:** 2026-06-27

**Authors:** Sylwia Głazowska, Helle J Martens, Bjarke Veierskov, Daniel P Persson

**Affiliations:** Department of Plant and Environmental Sciences, University of Copenhagen, Thorvaldsensvej 40, 1871 Frederiksberg C, Copenhagen, Denmark; Department of Geosciences and Natural Resource Management, University of Copenhagen, Rolighedsvej 23, 1958 Frederiksberg C, Copenhagen, Denmark; Department of Plant and Environmental Sciences, University of Copenhagen, Thorvaldsensvej 40, 1871 Frederiksberg C, Copenhagen, Denmark; Department of Biosystems and technology, Swedish University of Agricultural Sciences, 234 56 Alnarp, Sweden

**Keywords:** *Abies nordmanniana*, budbreak, calcium, homogalacturonan, magnesium, mineral deficiency, Nordmann fir, pectin, remobilization

## Abstract

The annual transport patterns of mineral nutrients in conifers remain poorly understood. Calcium (Ca) and magnesium (Mg) deficiencies often occur in planted conifer stands and cannot be easily remedied by simply adding these nutrients to soil. This study examined translocation and remobilization of Ca and Mg in young Nordmann fir (*Abies nordmanniana*) trees. Our data revealed that Ca and Mg absorbed by the roots do not reach the needles during the current growth season, indicating that internal nutrient remobilization is crucial for the growth of new needles, particularly shortly after the budbreak. Monitoring 1-year-old needles over a full annual cycle revealed steep declines in Ca and Mg around budbreak, coinciding with their increase in the emerging needles on the same branch. This strongly suggests that older needles supply Ca and Mg to new needles. Trees with low Mg concentration in 1-year-old needles in the spring developed visual Mg deficiency symptoms during the summer. For Ca, which is immobile in the phloem, we recorded a substantial increase in the apoplast of older needles just before budbreak. Simultaneously, the immunolocalization of cell wall homogalacturonan in these needles declined, suggesting pectin modification that facilitates Ca release from cell walls, enabling its translocation to the neighbouring, current-year needles. Our findings reveal a previously unknown process that advances understanding of mineral nutrient remobilization in conifer trees.

## Introduction

All perennials are subject to seasonal cycles that require modifications of their physiological functions in response to various environmental cues. The transition from winter dormancy to budbreak and the growth of new needles represents a major phenological event that strongly affects tree health via uptake, storage and remobilization of carbohydrates and mineral nutrients (Singh et al. 2017). Efficient transport of these resources is vital to sustain the new growth, since emerging needles show rapidly increasing respiration rates and cell division, creating a high demand for mineral nutrients (Myking 1998, Xie et al. 2020). All trees face significant challenges with fulfilling this need, particularly for phloem-immobile nutrients like calcium (Ca), which largely depend on transpiration for their transport (Malone et al. 2002, Paiva 2019). In mature trees, the distance between roots and current-year needles, combined with the high cation exchange capacity of xylem cell walls and radial nutrient losses to surrounding sink tissues, limits the efficiency of long-distance Ca transport (Kuhn et al. 1997, Pfautsch et al. 2015). In addition, vascular connections between buds and the rest of the tree are interrupted during winter to protect the bud from freezing damage and cavitation (Savage and Chuine 2021).

Together, these facts point towards a major role of remobilization of resources from neighbouring tissues, such as branches and mature needles during early spring growth. Indeed, 1-year-old needles may supply carbohydrates to their ‘neighbours’, i.e. current-year needles, thus emphasizing the importance of carbohydrate remobilization during early needle growth (Schädel et al. 2009). To what extent this remobilization also applies to mineral nutrients remains uncertain.

Nordmann fir (*Abies nordmanniana*) is an evergreen coniferous tree, popular in Christmas tree production, especially for the European market. It often develops foliar symptoms of Ca and Mg deficiencies, even in seemingly favourable environments, i.e., in fertile soils. Many studies have demonstrated that supplementary Ca and Mg soil fertilization has no immediate effect in remediating these deficiencies (Mitchell et al. 1999, Jandl et al. 2001), thus indicating that Ca and Mg deficiency symptoms in conifers may be a result of restrictions in uptake and/or translocation rather than low nutrient availability. We set out to investigate the transport and remobilization patterns of these mineral nutrients, one phloem-immobile (Ca) and the other phloem-mobile (Mg).

Calcium stabilizes membranes, mediates pectin cross-linking in cell walls and acts as a secondary messenger in multiple signalling pathways related to both growth and responses to environmental stresses (Lautner and Fromm 2010, de Bang et al. 2021). Poplar grown under Ca-starved conditions were incapable of responding to cold shock or wounding (Lautner and Fromm 2010). This highlights the importance of Ca in vital tree functions, both as a signalling molecule and as an element that affects the structural integrity of cell walls and woody tissues. Calcium can be cytotoxic even at moderate concentrations, thus it must mainly be stored outside the plant symplast (Borer et al. 2004).

Whereas symptoms of Ca deficiency in annual crops are well described (de Bang et al. 2021), they are typically less obvious in conifers. In red spruce, field studies demonstrated that low Ca concentrations in the needles increased winter injury (DeHayes et al. 1999). At concentrations below 800 mg Ca kg^−1^ DW, a significant correlation was observed between reduced cold hardiness and total foliar Ca (DeHayes et al. 1999). The same symptoms were reversed by Ca amendments, showing a relationship between Ca levels in needles and frost tolerance (Schaberg et al. 2011). In Nordmann fir, the Ca deficiency symptoms typically appear on young, current-year needles located on older whorls. The symptoms appear after shoot elongation, initially as yellow spots that gradually turn into large necrotic bands during the summer. The severity of the symptoms varies and is affected by environmental factors such as precipitation, elevation or shading. In Nordmann fir stands, Ca deficiency has been linked to the growth disorder known as Current Season Needle Necrosis (CSNN). Multiple foliar application of high concentration CaCl_2_ solutions reduced the severity of symptoms, but had a cytotoxic effect on the needles (Chastagner et al. 1990, Chastagner 1997). However, the exact underlying cause of CSNN remains unclear, since the fungus *Sydowia polyspora* has also been associated with this disorder (Talgø et al. 2010).

Magnesium is important for photosynthesis (Tränkner et al. 2018) and the regulation of Ca^2+^, K^+^ and H^+^ plasma membrane transporters (Shabala and Hariadi 2005). Magnesium is also essential for the synthesis of Rubisco and Rubisco activase (Tränkner et al. 2018). Contrary to Ca, Mg is mobile in the phloem and therefore less dependent on transpiration-driven transport. Magnesium deficiency in conifers, termed Upper Mid-Crown Yellowing (UMCY), typically appears as chlorosis on the distal half of older needles located on the whorls close to the main stem. Like CSNN, the processes leading to UMCY remain largely unclear (Beets et al. 2004).

Mineral nutrient transport into emerging needles represents a significant knowledge gap. To our knowledge, there is no detailed mechanistic explanation of how, when and from where low-transpiring, emerging conifer needles acquire an adequate supply of mineral nutrients. In this study, we did not detect any transport of Ca or Mg from the roots to the growing needles, but by measuring the seasonal changes in mineral concentrations in 1-year-old and current-year needles from the same branch, we found a substantial decrease of both Ca and Mg in the 1-year-old needles around the time of budbreak. Simultaneously, Ca and Mg concentrations increased in the current-year needles, suggesting remobilization from older to younger needles. While Mg remobilization was expected due to its phloem mobility, the substantial Ca remobilization was surprising. Since carbohydrates released from conifer cell walls are a plausible carbon source for emerging needles (Dickmann and Kozlowski 1970, Renault and Zwiazek 1997, Schädel et al. 2009), we specifically analysed the association between pectin and Ca in the cell walls of 1-year-old needles. Using advanced microscopy, immunohistochemistry and element bioimaging, we observed a substantial increase of Ca in the apoplast, which coincided with a distinct decrease in pectins with Ca binding capacity. Our results show that the pectin network in conifer cell walls acts as Ca storage, which can undergo dynamic alterations that release and transport Ca to sink tissues such as emerging needles. Our findings demonstrate a previously unrecognized mechanism for Ca remobilization during early spring growth in conifer trees.

## Materials and methods

### Histology

**Figure 1:**
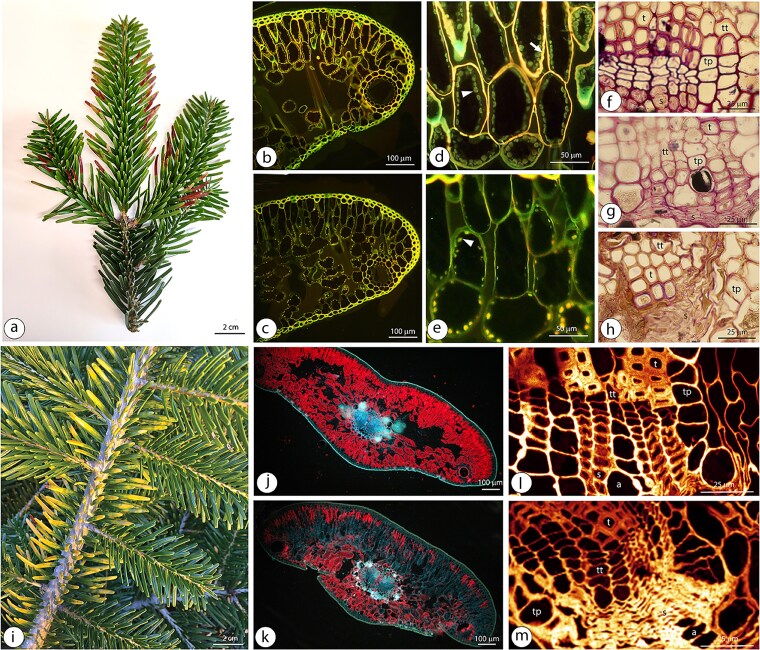
Symptoms and anatomical alterations associated with Current Season Needle Necrosis (CSNN) and Upper Mid-Crown Yellowing (UMCY) in needles of Nordmann fir (*Abies nordmanniana*). (a) Necrotic regions on current-year needles, characteristic of CSNN. (b and c) Coriphosphine-stained resin-embedded cross sections from needles with CSNN showing a healthy-looking green region (b) and a tissue adjacent to necrotic discolorations (c). (d and e) Close-up views of palisade mesophyll in the green region (d) and near the symptomatic tissue (e), chloroplasts (arrowheads) and pectin-rich cell walls (arrow) are indicated. (f–h) Astra blue and Safranin-stained resin-embedded sections of vascular tissue in a control needle (f), in the green region (g) and in the reddish, symptomatic region (h) of the CSNN-affected needle, showing collapsed phloem cells. (i) Partially chlorotic older needles within the whorl, characteristic of UMCY. (j and k) Cryo-sections showing chlorophyll autofluorescence (red): a control needle with strong autofluorescence in palisade and spongy mesophyll (j) and a chlorotic region of a UMCY-affected needle with reduced chlorophyll autofluorescence, particularly in the palisade mesophyll (k). (l and m) Representative images of Safranin-stained resin-embedded sections showing vascular tissue in a healthy needle (l) and visibly collapsed phloem cells in a chlorotic region of a UMCY-affected needle (m). a, Strasburger cells (albuminous cells); s, sieve cells; t, tracheids; tp, transfusion parenchyma; tt, transfusion tracheids.

Needles with clear symptoms of CSNN were harvested in late June from 6-year-old field-grown trees at HC Juletræer Lynge, Zealand (DK) (for site description, see Veierskov 2023) (Figure 1). Briefly, 2-mm-long needle pieces were fixed in Karnovsky’s fixative (4% (w/v) paraformaldehyde and 5% (v/v) glutaraldehyde in 0.1 M sodium cacodylate buffer, pH 7.3), post-fixed with 1% (v/v) OsO_4_ in 0.2 M cacodylate buffer, pH 7.3 and embedded in Spurr’s resin (Liesche et al. 2019). Sections (3 μm) were prepared using an ultramicrotome (EM UC7; Leica Microsystems), stained with 0.03% aq. Coriphosphine (Weis et al. 1988) and observed under a fluorescence microscope (Nikon Eclipse 80i). Other sections were first stained with 1% Astra Blue, then counterstained with 1% Safranin O, according to the protocol by Novikov and Sup-Novikova (2021) and observed using a Nikon Eclipse 80i microscope in brightfield mode.

Chlorotic needles affected by UMCY were sampled from stems of 3-year-old branches of potted 4-year-old plants grown at Kirstinebjerg nursery (Lolland, Denmark) (Figure 1). Needles with similar age and location on the tree were harvested from healthy trees as controls. Resin-embedded samples were prepared, sectioned and stained with Coriphosphine as described above. The sections were imaged using a Leica SP5 X confocal laser scanning microscope, using the following settings: argon laser excitation 458 nm line, emission 625–686 nm, 20× oil immersion objective and line average 8. A series of optical sections were collected (*xyz*-mode, line average 2, 16 μm size depth), and maximum-intensity projections were created in the LAS AF software. In addition, 8- to 10-mm-long pieces of fresh needles were placed in OCT medium (Optimal Cutting Temperature, Tissue-Tek from Sakura Finetek, Japan) and snap-frozen in liquid N_2_ (Persson et al. 2016). Then, 35-μm-thick sections were prepared using a cryotome (Leica model CM050S) and imaged directly with a Nikon Eclipse 80i FM microscope with V-2A UV excitation filter (380–420 nm) and LP450 emission filter.

### Plant cultivation

**Figure 2:**
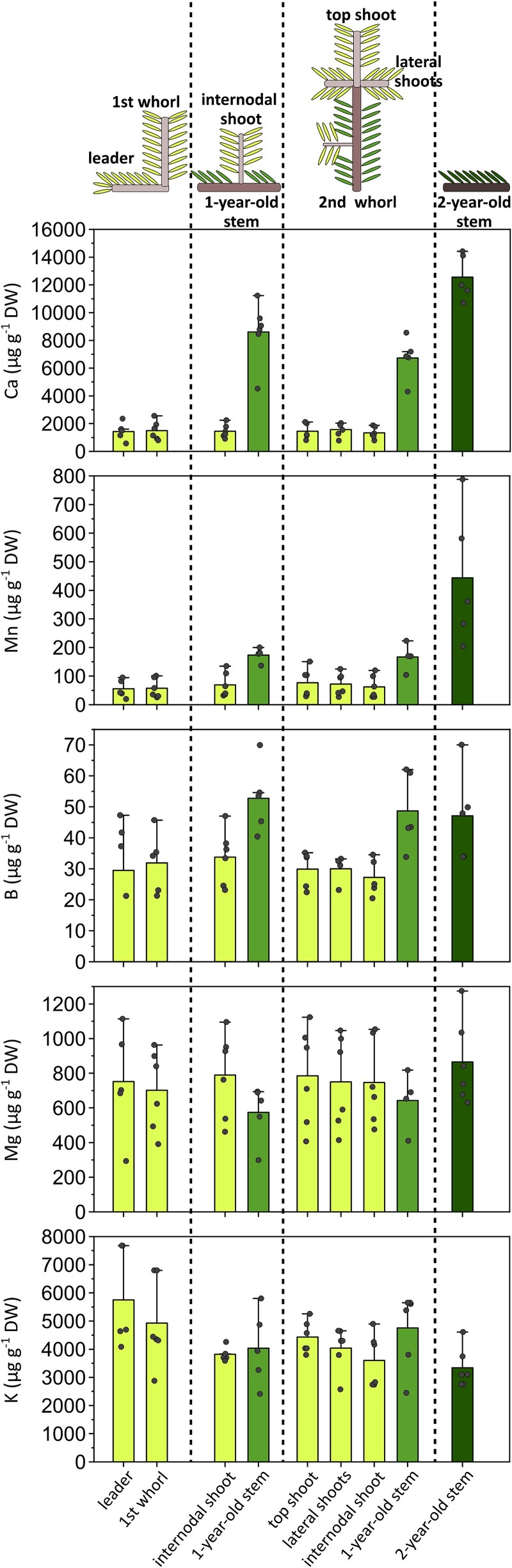
Concentrations of chosen phloem immobile (Ca, Mn, B) and mobile (Mg, K) elements in needles collected from different parts of Nordmann fir trees. Current-year (light green), 1-year-old (green) and 2-year-old needles (dark green) were harvested from different parts of a 3-year-old tree as presented in the scheme above. Bar charts present mean concentration values (±SD, *n* ≤ 6) of phloem immobile elements, Ca, Mn and B, as well as phloem mobile elements; Mg and K analysed using ICP-OES.

**Figure 3:**
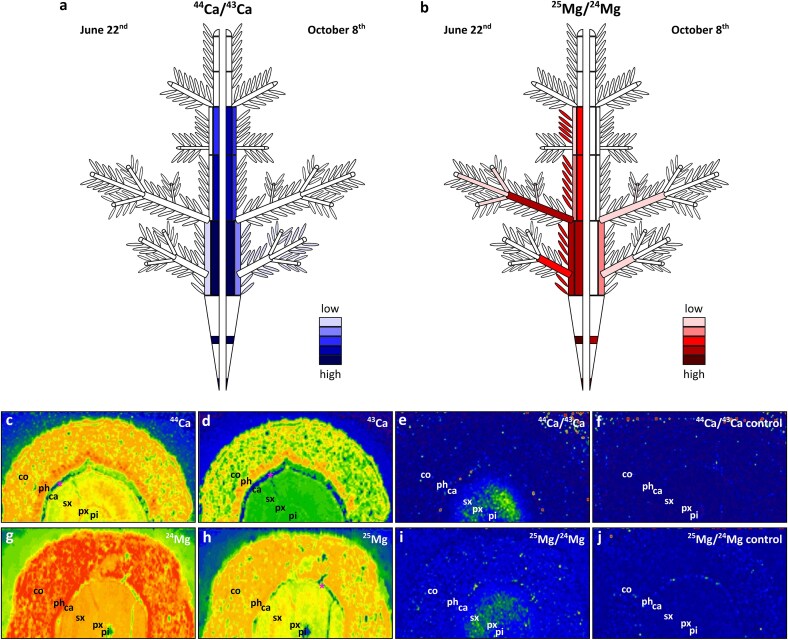
Calcium and Mg uptake and translocation in Nordmann fir. Upper figures (a and b): 3-year-old trees were supplied with isotope-enriched Ca (^44^Ca) and Mg (^25^Mg) and harvested at two time points after isotope addition (8 days [June samples]) and 3.5 months [October samples]). Deviation from the natural isotope ratios, ^44^Ca/^43^Ca (a) or ^25^Mg/^24^Mg (b), is presented as a heatmap, with dark colours corresponding to the highest and light colours to the lowest values. Bottom figures: LA-ICP-MS images of the isotope distribution and ratios in the lower stem of Nordmann fir harvested in June (8 days after isotope addition). Images show the distribution of ^44^Ca (c), ^43^Ca (d), ^24^Mg (g) and ^25^Mg (h) isotopes, as well as the ^44^Ca/^43^Ca and ^25^Mg/^24^Mg ratios in the treated plants (e and i) and in untreated controls (f and j). Asterisks indicate physical damage that occurred during drying of the samples. The various tissue types are indicated, where: pi, pith; px, primary xylem; sx, secondary xylem; ca, cambium; ph, phloem; co, cortex.

For the analysis of total concentrations of mineral elements in all plant parts (Figure 2; Figures S1 and S2 available as Supplementary Data at *Tree Physiology* Online) and for the isotope translocation studies (Figure 3a and b), 3-year-old plants were used, all potted in the fall and kept outside under a roof. All seedling plants were germinated and pre-cultivated as described by (Rasmussen et al. 2009). In the first week of April, 1 month prior to the start of experiments, the plants were uprooted and then thoroughly washed to remove all the soil. Then the plants were transferred to 2 L containers filled with sand. The sand was acid-washed in 35% HNO_3_ before planting, to remove any contaminating ions. After the acid wash, the sand was washed multiple times with Milli-Q water (Milli-Q Element; Millipore) until the pH approached neutral (pH ~6). Plants in the sand culture were watered weekly with a nutrient solution containing: 200 μM KH_2_PO_4_, 200 μM K_2_SO_4_, 300 μM MgSO_4_^.^7H_2_O, 100 μM of NaCl, 300 μM Mg(NO_3_)_2_∙6H_2_O, 900 μM Ca(NO_3_)_2_∙4H_2_O, 600 μM KNO_3_, 50 μM Fe(III)-EDTA Na, 1 μM MnCl∙4H_2_O, 0.8 μM Na_2_MoO_4_∙2H_2_O, 0.7 μM ZnCl_2_, 0.8 μM of CuSO_2_∙5H_2_O, 0.8 μM NiCl_2_, 2 μM H_3_BO_4_. All nutrient solutions were prepared in Milli-Q water.

### Total concentration of mineral nutrients

At harvest, each tree from the sand culture was parted into the following fractions: root tip (1 cm long fragment); root 15 cm from root tip (1-cm-long fragment), lowest above-ground stem section (2-year-old stems), ending at the junction with the first whorl of branches. These stem samples were then divided into core stem and stem bark, first whorl branches and needles, second stem section (between first and second whorl, again with core stem and stem bark), second whorl branches and needles and finally the top shoot with branches and needles.

Briefly, all samples were homogenized, then digested in a microwave oven using concentrated HNO_3_ (70%). All samples, including certified reference material (CRM1515 apple leaves, National Institute for Standards and Technology [NIST]), were digested in a pressurized digestion chamber (Ultrawave, Milestone Inc., Bergamo, Italy) at 240 °C for 10 min, with a reduced amount of ballast water for heat transfer. The full cycle consisted of 15 min ramping to the digestion temperature, maintenance of this temperature for 10 min and then cooling down for another 15 min, giving a total runtime of 40 min. After digestion, samples were diluted with Milli-Q water to a final acid concentration of 3.5% and then analysed by Inductively Coupled Plasma Optical Emission Spectroscopy (ICP-OES) (Chen et al. 2020).

### Stable isotope uptake and translocation

We used stable isotopes (^44^Ca and ^25^Mg, Trace Sciences International, Canada) to follow the uptake and translocation of Ca and Mg from roots to shoots. The isotopes were dissolved in 2.5 M HCl, diluted and mixed with the nutrient solution to a final concentration of 250 μM. Two days prior to isotope addition, the plants were starved for Ca and Mg, e.g., plants that were to receive Ca isotopes were given a nutrient solution without Ca, and plants that were to receive Mg isotopes were given a nutrient solution without Mg. The isotopes were added to all plants in early June, then a second time 5 days later. Half of the plants, e.g., two plants from each treatment (Ca or Mg isotopes), were harvested after 8 days, including two control plants. The second half of the plants (again two with Ca isotopes, two with Mg isotopes and two controls) were left growing until October, then harvested. All samples from the isotope experiment were prepared as described in the paragraph above but were analysed with an Inductive Coupled Plasma Mass Spectrometer (ICP-MS, model 7900, Agilent Technologies, USA), using the isotope ratio (IR) feature of the ICP-MS software. The enrichment was calculated by dividing the enriched isotope by a non-enriched isotope. In non-enriched samples, this gives values close to the theoretical isotope ratios (IR_t_): 15.45 for Ca and 0.127 for Mg (IR_t_: ^44^Ca = 2.086%, ^43^Ca = 0.135%, ^44^Ca/^43^Ca = 15.45, ^25^Mg = 10%, ^24^Mg = 78.99%, ^25^Mg/^24^Mg = 0.127). The use of the low-abundant ^43^Ca isotope was necessary because the most abundant isotope, ^40^Ca, is heavily interfered by the argon gas of the plasma. All untreated controls deviated less than 5% from the IR_t_, and samples with deviation <10% from untreated controls were not considered enriched.

### Element bioimaging in cryosections

Samples were prepared for element bioimaging by a Laser Ablation Inductively Coupled Plasma Mass Spectrometry system (LA-ICP-MS), as described in (Persson et al. 2016) (Figure 3; Figure S3 available as Supplementary Data at *Tree Physiology* Online). Briefly, each sample was placed in a mould made of aluminium foil, filled with OCT medium (Optimal Cutting Temperature, Tissue-Tek from Sakura Finetek, Japan). Hereafter, each sample was frozen in liquid nitrogen (N_2_) and then transferred to a pre-cooled (−25 °C) cryotome (Leica model CM050S) for sectioning. The thickness of the sections was 25 μm, and all samples were sectioned with the help of cryofilm according to the Kawamoto method (Kawamoto and Kawamoto 2014), using 2B cryotape (SectionLab, Tokyo, Japan). Prior to analysis, these samples were freeze-dried overnight.

Samples were analysed with an NWR 193 Laser (New Wave Research, USA), with the following key settings for the results in Figure 3: energy: 5.8 J cm^−2^, scan speed: 80 μm s^−1^, repetition rate: 60 Hz and spot size 20 μm. Elements were detected with an Agilent 7500ce ICP-MS operated in standard (no gas) mode and RF power: 1500 V, sample depth: 5.5 mm and carrier gas flow: 1.0 L min^−1^. The isotopes analysed were ^24^Mg, ^25^Mg, ^43^Ca and ^44^Ca. From the inside and out, the images display the pith, primary xylem, secondary xylem, cambium, phloem, cortex and outer bark. Please note that during drying of the samples, minor physical damage occurred between the cambium and the phloem (indicated with a*), which was not related to any biological factors.

### Eosin uptake and translocation

**Figure 4:**
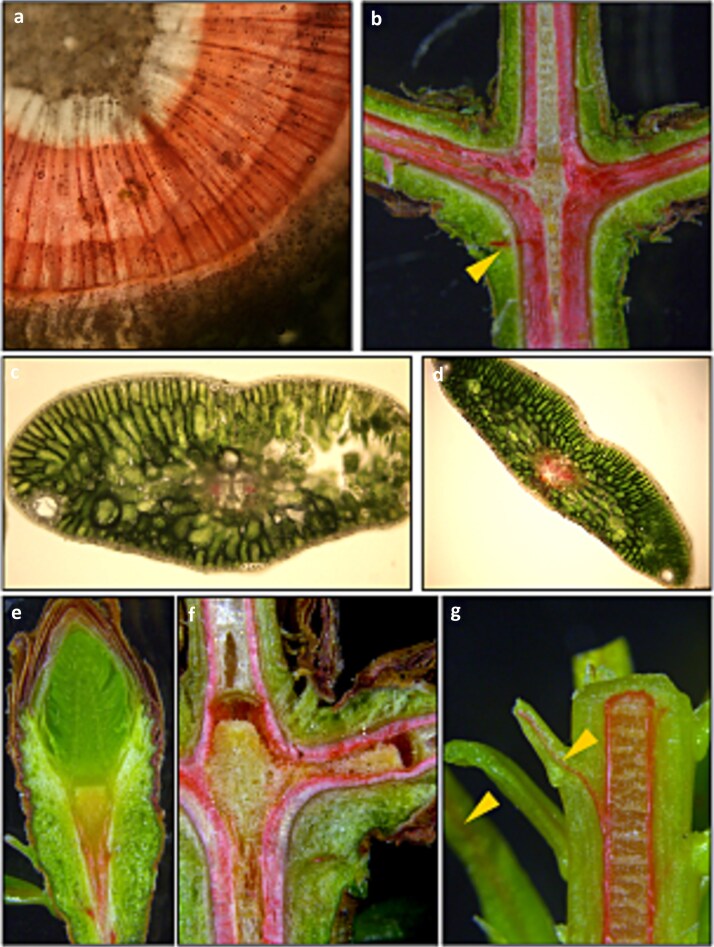
Eosin translocation into various organs of Nordmann fir. Eosin movement was traced in 3-year-old plants to study the functionality of the conductive tissues. The red dye was detected in the xylem throughout the basal part of the stem (~10 cm above the root system) (a) and in a longitudinal section through the branching point of the top whorl (b); it also moved into needles growing directly on the main stem (arrowhead). Cross sections through current-year (c) and 2-year-old needles (d), both harvested after budbreak, showed the presence of Eosin in the vascular bundle. Before budbreak, Eosin moved all the way to the leader, where the dye stopped at the crown barrier in buds (e). After budbreak, it moved freely into new growth (f), where it could be observed in current-year needles (arrowhead) (g).

Trees grown in sand cultures (see `Plant cultivation' section above) were uprooted, and the roots were washed and submerged in 0.5% aqueous Eosin solution (Figure 4). The container with roots was covered with aluminium foil and incubated at 23 °C and vapor pressure deficit between 0.025 and 0.029 kPa. During the first 3–4 h Eosin movement was monitored in needles from all parts of the tree, and after 24 h the whole trees were harvested. Then the roots were quickly dipped in MQ water to remove the excess Eosin and dried with a paper towel. The plants were divided into 3-cm-long segments, cross sections of the top and bottom parts of each segment were prepared, then the remaining part of the segment was cut in the middle following the longitudinal axis. In addition, needles of various ages were harvested from different parts of the tree, intact needles and cross sections were prepared. All segments were assessed using a fluorescence stereo microscope (Leica M205 FA) in brightfield mode.

### Total concentration of mineral nutrients in 1-year-old and current-year needles throughout the year

**Figure 5:**
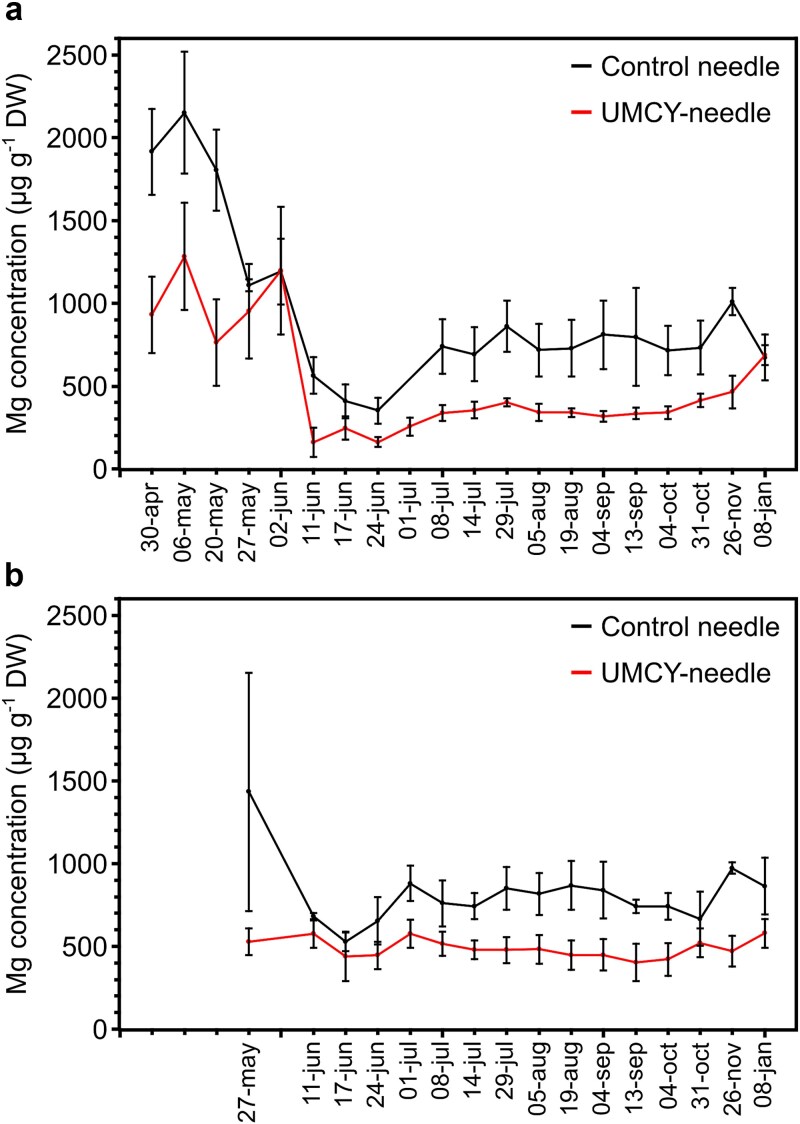
Concentrations of Mg in control and UMCY-needles throughout a year. Magnesium concentration was measured in (a) 1-year-old needles and (b) current-year needles, using ICP-OES. Charts present mean concentration values (±SD, *n* ≤ 3). Budbreak occurred between 20th and 27th of May.

**Figure 6:**
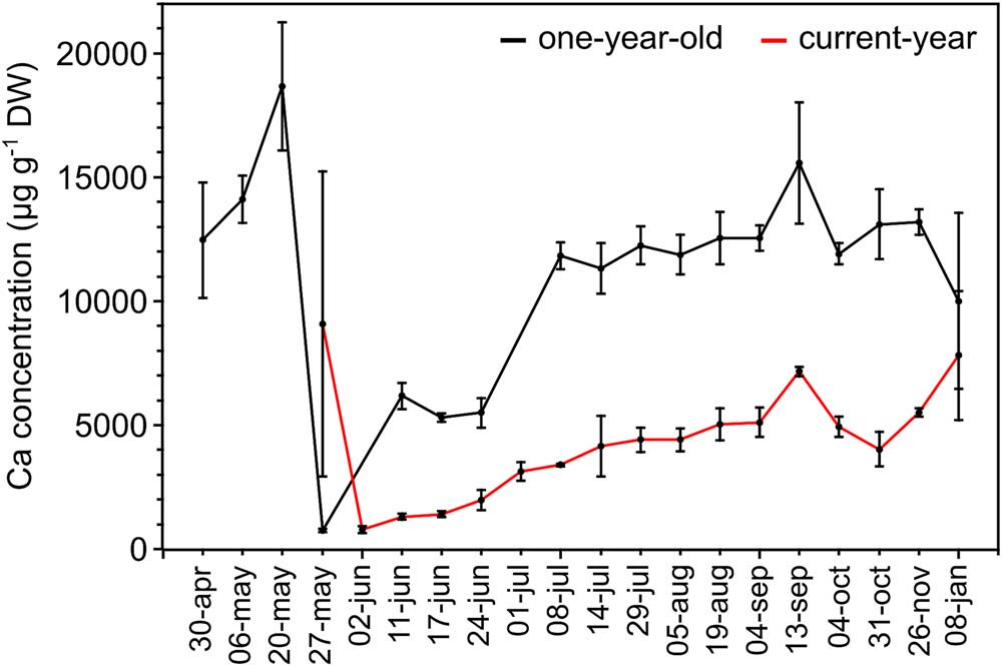
The Ca concentration in 1-year-old and current-year old needles of healthy control plants harvested during a year. Calcium concentration was measured using ICP-OES. Charts present mean concentration values (±SD, *n* ≤ 3). Budbreak occurred between 20th and 27th of May.

For the analysis of annual fluctuations of total mineral nutrient concentrations, we sampled needles from the shoot tip of the second whorl from the top on plants grown for 6 years in the field at HC Juletræer, in both control plants and in plants that developed UMCY symptoms later in the growth season (hereafter called UMCY-trees) (Figures 5 and 6; Figure  S4 available as Supplementary Data at *Tree Physiology* Online). There was no visible presence of pathogens on the selected trees that could have affected the development of the symptoms. Needles were continuously sampled from April to January the following year. Five trees with desired Christmas tree traits were selected as controls. Another 20 trees that had UMCY symptoms the previous year were selected, and needles were sampled as for control plants, but the samples were kept in a −60 °C freezer until UMCY symptoms appeared on some of the trees. Samples from five trees that developed UMCY symptoms were selected, and sampling continued from these trees only. From January, three needles were sampled from within 10 mm of the shoot tip on the second whorl. After budbreak, sampling continued from the old shoot, and for the first 3 weeks, all needles on the lower half of the new shoot were collected, whereafter five needles were collected from the lower 10 mm of the new shoot. All samples were prepared and analysed with ICP-OES as described above; all essential plant nutrients and strontium (Sr), a Ca analogue, were included in the analysis.

### Element bioimaging in resin-embedded sections

Approximately 5-mm-long pieces were cut from the middle part of the 1-year-old needle and fixed in Karnovsky’s buffer. Subsequently, these samples were washed in cacodylate buffer and dehydrated using an ethanol dilution series (30%, 50%, 70%, 96% and absolute ethanol). Absolute ethanol was then removed, and the samples were infiltrated with a mix of LR White (Agar Scientific, London, UK) with absolute ethanol (1:3, 1:1, 3:1), and finally placed in pure LR White, left overnight and polymerized at 60 °C for 48 h. The blocks were sectioned with an ultramicrotome (Leica EM-UC7) and a glass knife to produce 5 μm sections.

**Figure 7:**
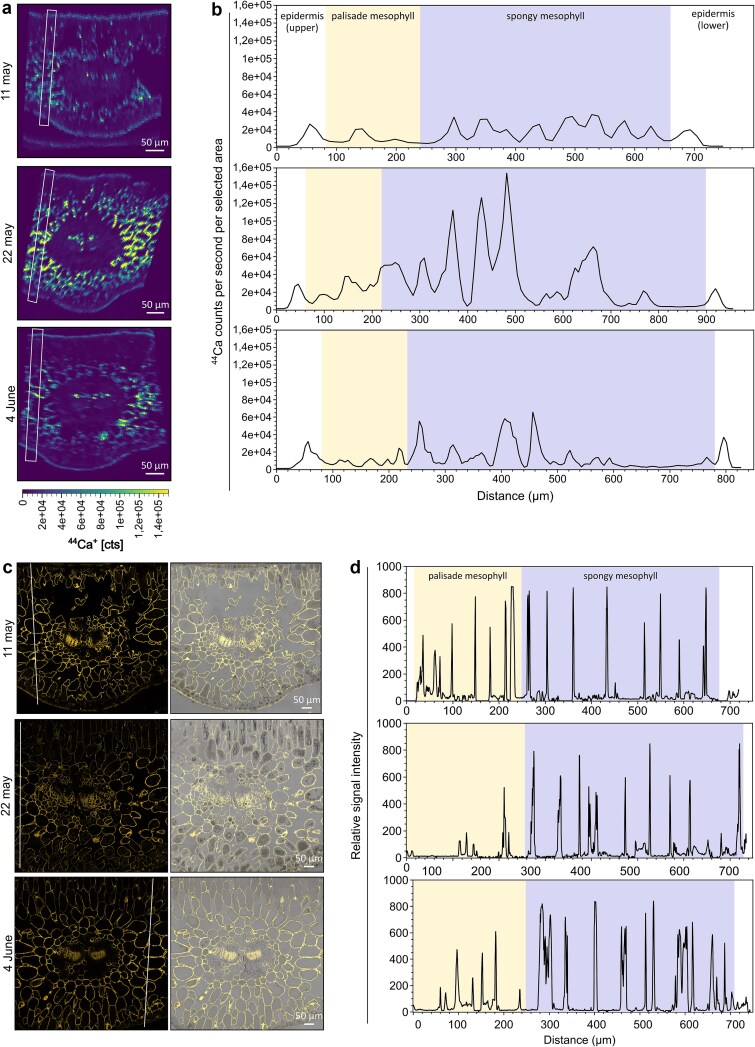
Calcium and pectin distribution in 1-year-old needles prior to, during and after budbreak. (a) Tissue mapping of ^44^Ca using LA-ICP-MS. Heatmaps present ^44^Ca distribution in cross-sections of resin-embedded 1-year-old needles (at three harvest times, presented in counts per second [cps]). (b) The average signal marked in panel (a) with white rectangles was displayed as a line graph. The marked area represents a view across all the needle tissues adjacent to the vascular bundle, i.e., epidermis and hypodermis on the upper side, palisade mesophyll (yellow colour), spongy mesophyll (lilac colour) and epidermis and hypodermis on the lower side of the needle. (c) Localization of homogalacturonan with a high degree of methylesterification detected using a specific monoclonal antibody (JIM7). (d) Line scan graphs showing the immunofluorescence intensity along the lines in panel (c); the profile presents signal across the whole needle in the tissues adjacent to the vascular bundle. Coloured boxes indicate palisade (yellow) and spongy mesophyll (lilac).

**Figure 8:**
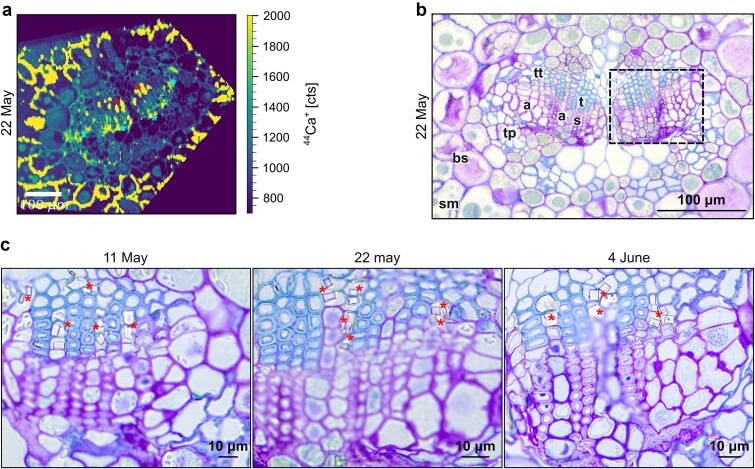
Calcium oxalate (CaOx) deposition in vascular bundles of 1-year-old needles. (a) ^44^Ca imaging in resin-embedded cross-sections of 1-year-old needles using LA-ICP-MS. The heatmap presents ^44^Ca distribution in counts per second (cps). Red asterisks indicate the ^44^Ca signal corresponding to the CaOx crystals shown in panel. Cyan asterisks indicate the ^44^Ca signal in the Strasburger cells, the transfusion parenchyma and the sieve cells. (b) Resin-embedded section stained with Toluidine Blue, showing the structure of a vascular bundle enclosed by a bundle sheath. (c) Close-up images of part of the vascular bundle (as indicated by a dashed rectangle in panel b) in samples harvested at three time points: before budbreak (11 May), during budbreak (22 May) and after budbreak (4 June). Red asterisks indicate deposition of CaOx crystals. a, Strasburger cells (albuminous cells); bs, bundle sheath; s, sieve cells; sm, spongy mesophyll; t, tracheids; tp, transfusion parenchyma; tt, transfusion tracheids.

The sections were analysed on a 193 nm ArF excimer laser (Iridia, Teledyne Photon Machines, Thousand Oaks, CA, USA) equipped with a Cobalt cell (Teledyne Photon Machines) and connected to an Inductively Coupled Mass Spectrometer (model 8900, Agilent Technologies, USA) (Figures 7 and 8; Figures S5 and S6 available as Supplementary Data at *Tree Physiology* Online). Analysis was done with the following laser settings: scan speed: 160 μm s^−1^, repetition rate: 200 Hz, fluence: 0.5 J cm^−2^ and spot sizes: 3–10 μm (Figure S4 available as Supplementary Data at *Tree Physiology* Online: 10 μm spot size, Figure 7: 5 μm and Figure 8: 3 μm). Helium (He) was used as transfer gas from the laser ablation unit to the ICP-MS with a total flow rate of 0.6 mL min^−1^. The ICP-MS was operated in standard mode with a sample cone depth of 30 mm and a nebulizer gas flow (Ar) of 0.75 mL min^−1^. Only ^24^Mg and ^44^Ca were analysed, in a scan cycle of 25 ms. Any drift in sensitivity was monitored before and after all samples, using a NIST612 glass standard (NIST, SC, USA). All images were processed with the High-Definition Image Processing software (HDIP, Teledyne CETAC Technologies), including gas blanks and signal drift corrections.

### Immunofluorescence localization of homogalacturonan epitopes

The resin blocks (see description above) were sectioned with an ultramicrotome (Leica EM-UC7) and a glass knife to produce 1 μm sections. The sections were blocked with 5% skimmed milk powder in PBS (MP/PBS) for 30 min, then probed with JIM7 primary antibody (1:10 dilution) in MP/PBS for 2 h and washed, then sections were probed for 2 h with anti-rat secondary antibody conjugated to Alexa555 (1:300 dilution; Thermo Fisher Scientific, Waltham, MA, USA) and washed tree times with PBS. Finally, Calcofluor White was added at a 1:100 dilution in PBS for 5 min and then washed with PBS. Samples were mounted in Citifluor (Agar Scientific, London, UK) and observed immediately. Confocal laser scanning microscopy was performed using an inverted microscope (Leica SP5) equipped with a 405 nm UV diode (for Calcofluor White) and helium/neon (543 nm) laser for AlexaFluor 555. Images were processed in LAS X (Leica) and line graphs were prepared using Fiji software (Schindelin et al. 2012) (Figure 7; Figure  S7 available as Supplementary Data at *Tree Physiology* Online). Each of the above-described probing experiments was repeated at least three times.

Sections were additionally stained with 0.02% Toluidine Blue O, which differentially stains polysaccharides and lignin and enables visualization of cells (Figure 8). Briefly, samples were incubated for 10 min, thoroughly washed with MQ and observed with a microscope (Leica DM 2000 LED) in brightfield mode.

## Results

### Impact of Ca and Mg deficiencies on the cellular structure of the needle

Current-year needles with red necrotic regions, distinctive CSNN symptoms (Figure 1a), were sampled within 2 weeks after the appearance of first visible symptoms. The green part of the symptomatic needles showed strong orange/yellow Coriphosphine labelling of mesophyll cell walls (Figure 1b), indicative of pectin-rich tissues. The staining was much weaker in the region adjacent to the red parts of the needle (Figure 1c), particularly in the cell walls of palisade mesophyll. Chloroplasts in this tissue also seemed smaller than in the controls (Figure 1d and e). Vascular tissue in the symptomatic needles was also severely affected, since co-staining with Safranin and Astra Blue showed weak and collapsed sieve cells in the green part of the CSNN-needle compared with the control needles (Figure 1f and g), which spread further to the transfusion parenchyma in the reddish, necrotic areas of the needles (Figure 1h).

A similar analysis was conducted for partially chlorotic needles with characteristic UMCY symptoms (Figure 1i), which were harvested from mid-July. Chlorophyll autofluorescence in the palisade mesophyll was strongly reduced in the symptomatic needles, compared with control needles (Figure 1j and k). Additionally, completely collapsed sieve cells were often seen in the chlorotic parts of UMCY-affected needles (Figure 1m) compared with control needles (Figure 1l).

### The nutrient concentration in needles of Nordmann fir is age-dependent

To better understand element distribution between different organs, 3-year-old trees were sectioned, and the stem and needles were divided into three age groups (2-year-old, 1-year-old and current-year; see `Total concentration of mineral nutrients' section of Materials and methods), and the element profile of each plant part was determined using ICP-OES (Figure 2; Figures S1 and S2 available as Supplementary Data at *Tree Physiology* Online). Needle age had a strong effect on concentrations, particularly for the phloem-immobile elements, including Ca, manganese (Mn) and boron (B) (Figure 2). The Ca concentration was particularly conserved within age group, irrespective of the sample location. Current-year needles harvested from different parts of the tree had about 5 and 8 times lower concentration than 1-year-old and 2-year-old needles, respectively. Similar patterns were observed for Mn and B, but the differences between age groups were less prominent.

Concentrations of Mg and potassium (K) showed more variation between biological replicates than between different needle ages, with no clear pattern between the needles of various ages (Figure 2).

### Calcium and Mg were taken up, but not translocated to needles

To trace the uptake and translocation of Ca and Mg, we supplied enriched stable isotopes (^44^Ca and ^25^Mg) to 3-year-old trees in mid-June, ~2 weeks after budbreak. We collected the samples at two time points to assess the speed of the process: 8 days after isotope addition to the growth media (short-term; June samples) and after 3.5 months (long-term; October samples). At harvest, each tree was subdivided into several fractions, and each of them was analysed for isotope enrichment (Figure 3a and b). The enrichment was calculated relative to the IRs measured in untreated plants (see ‘Stable isotope uptake and translocation’).

Roots consistently had the highest ^44^Ca (Figure 3a) and ^25^Mg (Figure 3b) enrichment, with a clear accumulation both at the root tip and in more differentiated root regions. In the short-term experiment (June samples), the ^44^Ca enrichment in all root samples was three- to six fold higher than in control plants, and for ^25^Mg it was approximately fivefold higher. At the same time, upward translocation of both isotopes was limited and declined gradually with increasing tree height. The ^44^Ca accumulation in the above-ground parts was mostly restricted to the main stem, and in the lowest, 2-year-old stem, the enrichment was ~10 times lower than in the roots (27–56% deviation from controls). The bark of this stem section showed only minor ^44^Ca enrichment (12%), while no enrichment was measured in needles or branches. Slightly more widespread enrichment was observed for ^25^Mg, where, in addition to the main stem and the bark, the isotope was detected in the stem of two lower branches and in needles sitting directly on the lowest and middle stem sections. The leader, first whorl (current-year) and all current-year needles had no isotope enrichment.

In the long-term experiment (October samples), roots showed the highest enrichment of both isotopes, with 8.5- to 16-fold ^44^Ca and 0.5- to 5.6-fold ^25^Mg enrichment, respectively (Figure 3a and b). Similar to the short-term study, enrichment declined with tree height.

The spatial distribution of the added isotopes in the lower stem sections from the short-term experiment (June) was investigated with LA-ICP-MS (Figure 3c–j). The untreated control showed no deviation from the natural isotope distribution (IR_t_), thus producing blank images (Figure 3f and j). In treated plants, both ^44^Ca and ^25^Mg enrichment were restricted to the inner parts of the stem, mainly parts of primary and secondary xylem (Figure 3e and i), which supports the results in Figure 3a and b. No isotope enrichment was detected in cambium, phloem or cortex, although these tissues showed the highest overall concentrations of Ca (cambium, phloem) and Mg (cortex, phloem).

To test whether the restricted isotope translocation towards the top of the trees was caused by low transpiration or obstructed xylem transport, Eosin dye was supplied to the roots of trees cultivated in an identical way as the trees in the short-term experiment (Figure 4). Eosin is a water-soluble dye that serves as a tracer for water movement through xylem (Katz et al. 1989). Within a few hours, Eosin moved to the top of the leader. The staining was observed in the xylem tissues of the main stem (Figure 4a), all the whorls (Figure 4b) and needles of different ages (Figure 4c and d), indicating unobstructed xylem conductivity. In an additional set of plants, Eosin dye was added before budbreak. Here, the dye stopped at the crown barrier in buds (Figure 4e) but moved into new growth soon after budbreak (Figure 4f and g), thus confirming that the vascular tissues of Nordmann fir are blocked between twigs and the bud prior to budbreak.

### One-year-old needles dramatically reduce their mineral element content during budbreak

Given the limited contribution of root uptake and xylem transport to new growth (Figure 3a and b), we hypothesized that current-year needles must depend heavily on the remobilization of Ca and Mg from older tissues. Therefore, we sampled 1-year-old needles continuously from April to January in both control plants and in plants that developed UMCY symptoms later in the growing season (hereafter called UMCY-trees) (Figure 5a). The current-year needles were sampled from late May (Figure 5c). In 1-year-old needles from the healthy trees, Mg concentrations were ~2200 μg g^−1^ dry weight (DW) in early May and then declined steadily for about 6–7 weeks (Figure 5a). Around budbreak, the concentration was 1100 μg g^−1^ DW but continued to decline to 350 μg g^−1^ DW by the end of June. Concentrations then recovered slightly during the late summer, stabilizing at ~720–800 μg g^−1^ DW during the autumn. The 1-year-old needles of the UMCY-trees consistently had lower Mg concentration than those of the control plants but showed similar decline and recovery period. However, the concentrations by late summer/fall never reached above 350 μg g^−1^.

In current-year needles of control trees (Figure 5b), Mg concentrations increased slightly from mid-June to early July and stabilized around 800–850 μg g^−1^ DW over the summer and autumn months. In the UMCY-trees, Mg concentrations in current-year needles were constantly lower than in the control samples, and the values remained just below 500 μg g^−1^ DW during the summer and autumn months.

Additionally, Ca concentrations were determined in current-year and 1-year-old needles of the healthy control plants (Figure 6; Table S1 available as Supplementary Data at *Tree Physiology* Online). In 1-year-old needles, the concentration increased from ~12000 to 18000 μg g^−1^ DW during the first weeks of May. Then, during budbreak, the average concentration declined sharply to ~700 μg g^−1^ within a single week (>95% decrease). Even though the individual trees analysed varied in their Ca concentration, the magnitude of the decrease was very consistent between the trees (95%, 96% and 96%) (see Table S1 available as Supplementary Data at *Tree Physiology* Online with data distribution for individual trees). In the weeks following budbreak, concentrations increased again, stabilizing at ~12000 μg g^−1^ DW by early July. In current-year needles, Ca concentration increased steadily during the summer, from 1300 to 7000 μg g^−1^ by mid-September.

The rate of concentration decline in 1-year-old needles was markedly different between Mg and Ca. Whereas the Mg concentration started to decrease in early May and continued gradually for almost 2 months, Ca declined abruptly between May 20 and 27 May, reaching its lowest values within a single week. The lowest Ca concentration occurred very close to budbreak. Concentrations of other mineral nutrients decreased around dramatically during May–June (Figure S4 available as Supplementary Data at *Tree Physiology* Online); all showed over 75% decline in their concentration around the time of budbreak, regardless of their phloem mobility. The timeline of concentration decline, however, varied between elements and spanned from 7 days to over 2 months (Figure S4 available as Supplementary Data at *Tree Physiology* Online).

### Calcium is released to the apoplast just prior to budbreak

LA-ICP-MS was used to further investigate the dynamics and spatial distribution of Mg and Ca in healthy 1-year-old needles before and after budbreak. As shown in Figure S3 available as Supplementary Data at *Tree Physiology* Online, Ca and Mg concentrations were substantially higher before budbreak than after, consistent with the total element concentrations (Figures 5a and 6). Since this sampling protocol preserves all basic elements in their native state (labile and bound) and localization (Persson et al. 2016), this result confirms that large quantities of both Mg and Ca are transported out of 1-year-old needles during budbreak. Magnesium shows a general decrease in all the tissues, while Ca has an ‘outside-in’ reduction, primarily in the palisade and spongy mesophyll closest to the epidermis (Figure S3 available as Supplementary Data at *Tree Physiology* Online).

To capture the dynamics at higher resolution, a second set of 1-year-old needles was analysed at three time points: before budbreak (11 May), during budbreak (22 May) and after budbreak (4 June). For combined microscopy and element bioimaging, these samples were embedded in resin and sectioned using an ultramicrotome. In contrast to snap-frozen samples (Figure S3 available as Supplementary Data at *Tree Physiology* Online), these mainly represent bound mineral nutrients, due to multiple washing and dehydration steps during sample preparation. LA-ICP-MS imaging (Figure 7a and b; Figure S5 available as Supplementary Data at *Tree Physiology* Online) supported that Ca is redistributed during the budbreak period. On 22 May, large quantities of Ca accumulated in the apoplastic regions of the spongy mesophyll surrounding the endodermis (Figure 7a), with the strongest signals concentrated around the vascular bundle. After budbreak (4 June), Ca signals had decreased substantially (Figure 7 and Figure  S5 available as Supplementary Data at *Tree Physiology* Online). The strongest Ca signal was always observed in the apoplast of mesophyll, but during budbreak, a high signal was also present inside the vascular tissues, including xylem, transfusion cells, Strasburger cells and phloem (Figure 8a). This observation suggests that Ca was mobilized to the apoplast from its binding sites in the tissue and then exported out of the needle.

The Mg signal was recorded in the same samples (Figure S6 available as Supplementary Data at *Tree Physiology* Online), showing only minor changes from before to after budbreak. This suggests that a majority of Mg exists in labile forms, which were washed away during the sample preparation. Hence, in contrast to Ca, Mg in these images likely represents a non-dynamic, residual Mg fraction.

### Pectin alterations coincided with the Ca release

Since we observed a strong Ca signal in the apoplast of mesophyll (Figure 7a), we decided to investigate the distribution of homogalacturonan (HG), an abundant cell wall component capable of binding Ca, in the same region. We used a specific monoclonal antibody (JIM7) recognizing methylesterified HG. Before budbreak, the JIM7 signal was evenly distributed throughout all the tissues (Figure 7c and d; Figure S7 available as Supplementary Data at *Tree Physiology* Online), but at budbreak (22 May) the signal dropped substantially, particularly in the palisade mesophyll and in the spongy mesophyll at the abaxial side around stomata (Figure 7c and d; Figure S7 available as Supplementary Data at *Tree Physiology* Online). After budbreak (4 June), the signal in the palisade mesophyll and spongy mesophyll was partly restored again, however with slightly lower intensities than before the budbreak (Figure 7c and d; Figure S7 available as Supplementary Data at *Tree Physiology* Online).

Together, these analyses suggest that Ca release from 1-year-old needles coincided with dynamic modifications of both pectin amount and structure. These modifications reduce the Ca-binding capacity in cell walls of specific mesophyll tissues, thereby enabling remobilization towards developing needles.

### Ca oxalates were detected in the vascular bundles of 1-year-old needles

We detected a strong Ca signal in vascular bundles of 1-year-old needles, especially around budbreak (Figures 7a and 8a); however, it did not always correspond with the pectin signal (Figure 7c). To further investigate, we examined Toluidine Blue-stained cross-sections for CaOx deposits. The crystals were observed inside the vascular bundle at all harvest times, both before and after budbreak (Figure 8b and c). Specifically, they were mainly present inside transfusion tracheids and xylem rays as single deposits or in groups of three to four crystals (Figure 8c), colocalizing with the strong Ca signal (Figure 8a). In contrast, the Ca signal seen in Strasburger cells, transfusion parenchyma and sieve cells (Figures 7a and 8a) most likely originates from the cell-wall-associated form or ions translocated towards xylem and phloem.

## Discussion

### Nutrient translocation limitation in conifers

Conifers face a range of inherent challenges in supplying nutrients to rapidly growing needles. These challenges include (i) long transport distance from roots to needles, (ii) nutrient depletion of the xylem sap along the way (Kuhn et al. 1997) and (iii) underdeveloped vascular tissues during early needle expansion (Savage and Chuine 2021). Our stable isotope labelling experiments revealed that, although Ca and Mg are taken up by roots, they are not translocated to current-year needles during the peak growth season (Figure 3a and b), despite a fully functional xylem (Figure 4). While water flows relatively freely through narrow xylem tracheids, divalent cations like Ca and Mg strongly bind to negatively charged groups in the cell wall, including hydroxyl, ether and carboxyl groups, which create a high cation exchange capacity in the xylem (Kuhn et al. 1997). This process slows ion movement relative to that of water (Lucas et al. 2013). In addition, radial nutrient transfer into surrounding tissues, including the cambium, a well-known accumulation site for Ca, Mg and K, may further reduce the ion flow towards distal sinks (Kuhn et al. 1997). During dormancy, the vascular connections between the buds and the branch are disrupted and are only gradually re-established basipetally during early needle expansion (Xie et al. 2020, Savage and Chuine 2021). These factors collectively limit direct xylem-mediated supply of Ca and Mg to developing needles during early spring growth.

### Nutrient remobilization from mature needles

Given the restricted root-to-needle transport, we focused on nutrient fluxes between 1-year-old and current-year needles. Historically, nutrient remobilization in conifers has been associated with nutrient conservation during senescence, primarily based on comparisons of living tissues with litter (Nambiar and Fife 1991). However, recent analyses of living tissue reveal that mature needles can substantially supply nutrients to growing needles (Nambiar and Fife 1991). For example, up to 60% of the phosphorus (P) in new needles of *Pinus radiata* originates from mature needles and the stem (Turner and Lambert 1986), with a comparable contribution reported for nitrogen (N) (Nambiar and Fife 1991, Vergutz et al. 2012). Similarly, in Norway spruce (*Picea abies*), twig xylem sap contained higher Ca, Mg, K and P fluxes than trunk sap (Dambrine et al. 1995).

Nutrient remobilization efficiency varies with tree age, site quality and climatic factors (de las Heras et al. 2017). In *Pinus sylvestris*, remobilization of N, K and Mg accounted for 83%, 82% and 52%, respectively, of all nutrients supplied to new shoot growth (Proe et al. 2000). Other studies using ^15^N, ^26^Mg and ^42^Ca isotopes injected directly into the sapwood of *Pinus pinaster* indicate long lag times for Ca and Mg transport into the needles: ^15^N reached the foliage within 2 weeks, whereas the ^26^Mg and ^42^Ca remained undetected in the needles for over a year (Augusto et al. 2011). Our data show a dramatic decrease of most of the essential mineral nutrients in 1-year-old needles around budbreak (Figures 5 and 6; Figure S4 available as Supplementary Data at *Tree Physiology* Online), consistent with similar seasonal fluctuations (N, P, K, Ca, Mg and B) reported in other conifer species, including Douglas-fir (*Pseudotsuga menziesii*), Fraser fir (*Abies fraseri*), Grand fir (*Abies grandis*), Noble fir (*Abies procera*) and Turkish fir (*Abies bornmuelleriana*) (Hart et al. 2012). Similar to our study, this study documented differences in the speed of nutrient decline in 1-year-old needles (1–8 weeks) (Hart et al. 2012). The authors did not explain the cause of these differences, but concluded that the time point and age of needles strongly influence nutrient concentration (Hart et al. 2012). All these reports are consistent with our findings, namely that mature needles, rather than roots, provide the primary source of mineral nutrients for emerging tissues in the early spring and suggest that such remobilization is a common strategy among conifers. In addition, our study highlights that suboptimal nutritional status in 1-year-old needles has dire consequences for emerging needles, as shown by the development of UMCY symptoms in trees with suboptimal Mg status (Figure 5).

### Calcium speciation and cell wall dynamics

Calcium remobilization is generally considered negligible due to its phloem immobility; thus, Ca typically accumulates in transpiring tissues over time (DeHayes et al. 1997, Paiva 2019, Malone et al. 2002). Additionally, its distribution is strongly influenced by its speciation and binding within plant tissues. In conifers, a large Ca fraction, possibly as much as 90% of the total Ca content (Fink 1991), is sequestered as poorly soluble CaOx complexes. However, it is still debatable whether CaOx solely protects plants from the cytotoxic effect of excess Ca or if it also serves as an active Ca storage, as both increasing CaOx concentration with needle age and decreasing CaOx during Ca deficiency, developmental maturation and drought have been reported (DeHayes et al. 1997, Franceschi and Nakata 2005, Paiva 2019). We observed crystal deposits within the vascular bundles of 1-year-old needles; however, at the given resolution, their amount appeared not to be affected by the high Ca demand of emerging current-year needles, indicating that these large CaOx deposits possibly do not play a significant storage role in Nordmann fir during budbreak and needle elongation (Figure 8b and c). However, we cannot completely rule out a role of CaOx in the Ca dynamics, especially the pool with a size below the resolution of light microscopy.

Calcium is also immobilized in the apoplast as part of the middle lamella and cell wall network through crosslinking of HG polymers (Obomighie et al. 2025), and this is likely a more soluble Ca fraction. Homogalacturonan is the most abundant type of pectin, and it consists of an unbranched α-1,4-linked d-galacturonic acid (GalA) backbone with various degrees of methylesterification and/or acetylation. If HG has a low degree of methylesterification, it also has a high capacity for Ca binding, contrary to highly methylesterified HG (Mohnen 2008). The HGs are synthesized as highly methylesterified forms (Knox et al. 1990, Mohnen 2008), which can be enzymatically modified to less methylesterified forms once deposited in the cell wall. Calcium-mediated crosslinking reduces cell wall porosity, leading to swelling and either decreased or completely restricted permeability of molecules, thus affecting not only water movement but also the Ca-binding capacity of the cell wall (Baron-Epel et al. 1988, Read and Bacic 1996, Obomighie et al. 2025). In buds, changes in HG methylesterification and Ca^2+^ dynamics in the so-called crown barrier have been linked to dormancy release (Lee et al. 2017). Our observations suggest that a similar mechanism occurs in 1-year-old needles (Figure 7c and d; Figure S7 available as Supplementary Data at *Tree Physiology* Online). Prior to budbreak, structure and/or amount of HG shifts, which coincide with a strong increase in apoplastic Ca. We suggest that alterations in the Ca-binding capacity of HG, especially in the mesophyll tissues close to epidermis, release Ca that can then be transported to emerging needles. This provides a mechanistic explanation for the previously observed Ca remobilization in evergreen conifers (Hart et al. 2012). Further work focusing on gene expression and activity of pectin modifying enzymes and cell wall profiling including detailed analysis of various pectin epitopes (both high- and low-methylesterification degree) would be extremely valuable for unravelling the temporal mechanism of the Ca release during budbreak.

### Potential transport pathways between needles

The exact transport mechanism for both Ca and other mineral nutrients remains unresolved, particularly at the early stages of budbreak. Phloem transport is unlikely for Ca due to its immobility, and transpiration-driven xylem flow may be insufficient early in needle expansion. Still, during bud flush and the beginning of cell division, Ca was reported to increase significantly in the apical meristem (Lautner and Fromm 2010). Several studies suggest that xylem can facilitate bidirectional transport of non-structural carbohydrates (NSCs) and potentially also minerals, even when transpiration is low. Such transport may be driven by water potential gradients rather than transpiration (Pfautsch et al. 2015, Tixier et al. 2017, Savage and Chuine 2021). In the walnut tree (*Juglans regia*), xylem was proposed as a major transport route of NSCs to developing buds, whereas the phloem maintained the necessary water circulation via Münch flow (Tixier et al. 2017). In addition, many studies have shown that structural carbohydrates released from cell walls of conifers are used as carbon source for emerging needles (Dickmann and Kozlowski 1970, Renault and Zwiazek 1997, Schädel et al. 2009), thus suggesting a link between cell wall modifications and remobilization of carbohydrates to emerging needles prior to their photosynthetic independence, possibly by gradient-driven xylem transport (Tixier et al. 2017). Indeed, any mineral nutrient loaded to the apoplast or xylem, including Ca, would be able to follow the water flow generated by a decrease in water potential. The xylem parenchyma cells apparently play a crucial role in this context, both for starch storage during dormancy and for its conversion into soluble carbohydrates that can be released into the xylem during early spring growth (Alves et al. 2007). In the xylem of walnut branches, large amounts of sucrose and hexoses were found in xylem, along with high activity of H^+^ATPases in the apical part of the branches, suggesting a proton-motif force for xylem loading (Alves et al. 2007). In support of this, xylem acidification occurs during spring and summer, but not during winter, thus aligning with the growth of new tissues (Pramsohler et al. 2022).

Even if many questions remain unanswered regarding the mechanism of remobilization in conifers, the interpretation of our data and a thorough examination of the existing literature permit us to suggest a conceptual model: (i) increasing day length and temperatures trigger budbreak via hormonal activation, (ii) vascular connections between bud and branch are re-established during budbreak as demand for carbohydrates and mineral nutrients increases in emerging needles, (iii) at the same time, structure and amount of HG in 1-year-old needles becomes altered, leading to release of Ca into the apoplast of mesophyll tissues, (iv) finally, available mineral nutrients, including Ca, are remobilized towards emerging needles via a currently unknown transport mechanism.

Our results warrant further studies on these transport pathways, specifically to elucidate how phenological events like budbreak and reactivation of vascular connections interplay with the remobilization of both carbohydrates and mineral nutrients. Such investigations should focus on whether the remobilization of carbohydrates is synchronized with the observed release of Ca or if it is a consequence of the same.

### Practical implications and future directions

Our findings indicate that Ca and Mg deficiencies in Nordmann fir are difficult to remediate with soil fertilization alone, due to limited direct root-to-needle transport during the growth season. Application of complementary foliar fertilizer solutions, optimized for penetration through the hydrophobic needle surface, may be a more effective way to deliver nutrients directly into needles, particularly in trees with early symptoms of mineral deficiency. Foliar fertilization of Nordmann fir is challenging and not well developed, thus approaches, including optimization of foliar formulations, potentially using biodegradable surfactants or the use of nutrient-loaded liposomes, should be considered.

Our data also consolidate the importance of sampling time when assessing the mineral nutrient status of needles. Seasonal fluctuations driven by remobilization can create a highly misleading snapshot of the nutrient content if samples are not carefully timed relative to phenological events, e.g., budbreak. Based on our findings, we suggest that the mineral nutrient status in 1-year-old needles in late April, at the beginning of remobilization, could be a good indicator of the true nutrient status of conifers. Another important implication is that conifer trees that do not have adequate amounts of mineral nutrients available for remobilization may develop deficiency symptoms in the current or following growing season. Thus, early spring, before budbreak, could be a suitable time for prophylactic foliar fertilization to provide older needles with sufficient amounts of mineral nutrients.

## Conclusions

In summary, we demonstrated that 1-year-old needles serve as an active source of the majority of mineral nutrients for emerging needles in Nordman fir, including both phloem-mobile (Mg) and phloem-immobile (Ca) nutrients. Seasonal fluctuations in nutrient content, combined with changes in HG-mediated Ca-binding capacity, suggest a dynamic remobilization mechanism. Understanding these processes have both fundamental and applied significance, from improving models of mineral nutrient cycling in conifers to designing effective strategies for foliar fertilization.

## Supplementary Material

Tree_Physio_Nordmann_fir_Supplementary_Figures_and_table_tpag064

## Data Availability

All data supporting the findings of this study are available from the corresponding authors upon request.
