# Supplementary material for: Good neighbours: current-year needles in Nordmann fir rely on their 1-year-old neighbouring needles for adequate nutrient supply
Source: Tree Physiol. 2026 Jun 27;46(7):tpag064. doi: 10.1093/treephys/tpag064 (PMC13358879; doi:10.1093/treephys/tpag064)
Supplement: Tree_Physio_Nordmann_fir_Supplementary_Figures_and_table_tpag064 [file tree_physio_nordmann_fir_supplementary_figures_and_table_tpag064.docx]

**
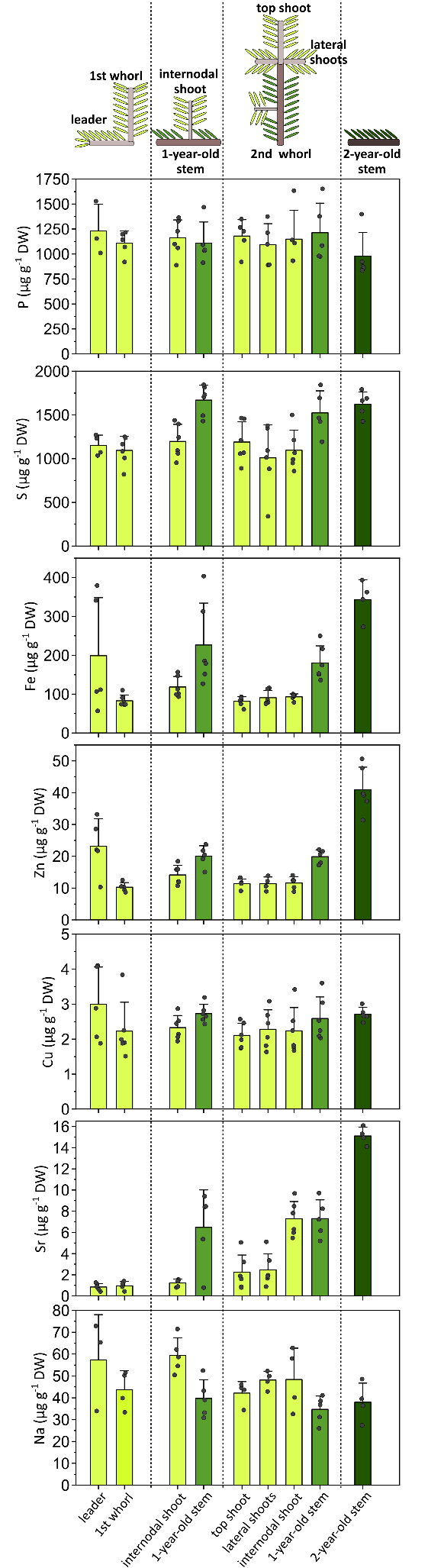
**

**Figure S1. Concentrations of elements in needles collected from different parts of the tree.**

Current-year (light green), one-year-old (medium green) and two-year-old needles (dark green) were harvested from different parts of three-year-old tree as presented on the scheme above. Bar charts present mean concentration values (±SD, *n ≤* 6) analysed using ICP-OES.

**
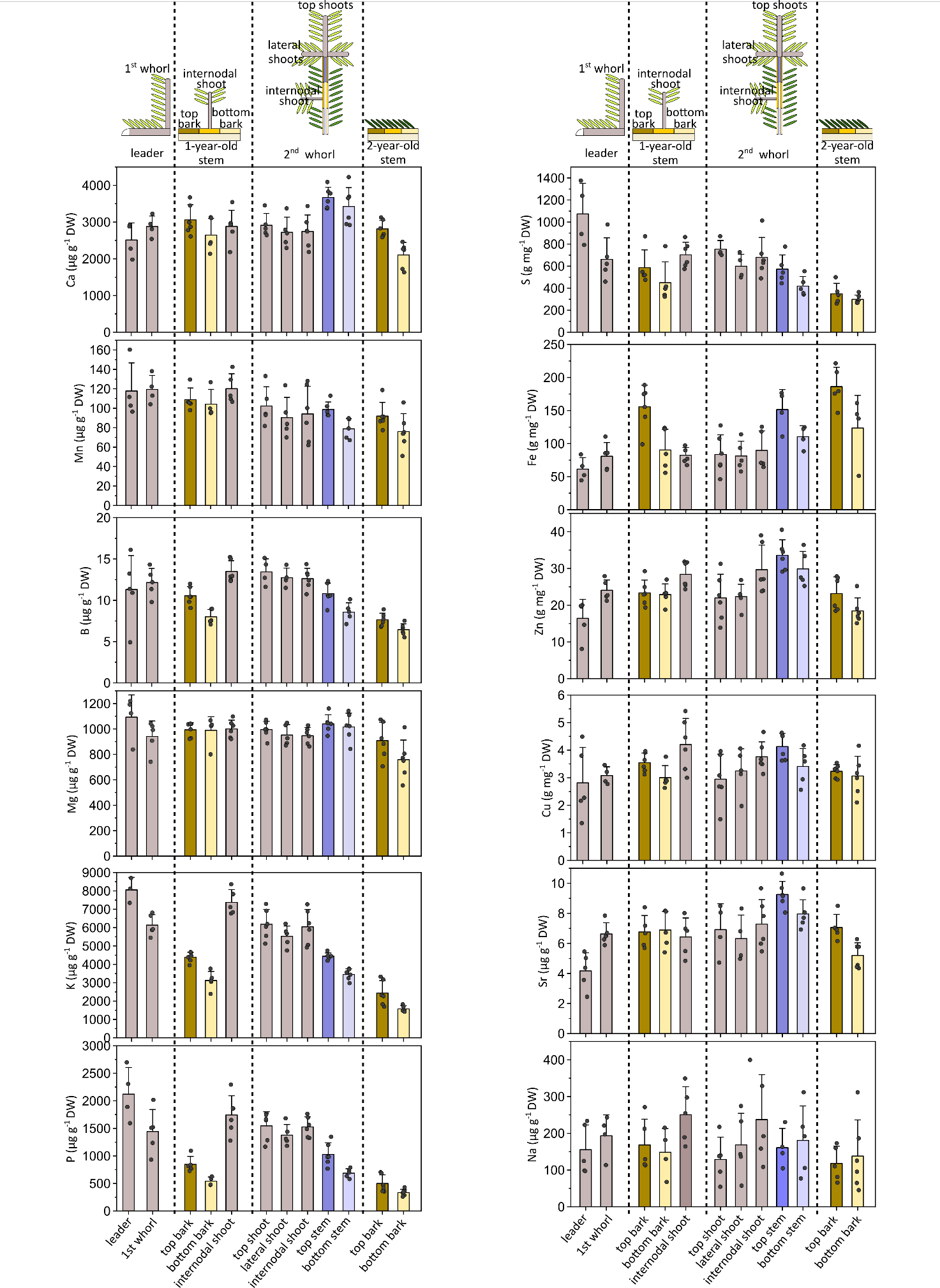
**

**Figure S2. Concentrations of elements in stem and bark of Nordmann fir trees.**

Stems and bark of three-year-old trees were divided as presented in the scheme above. Bar charts present mean concentration values (±SD, *n ≤* 6) of analysed using ICP-OES.

**
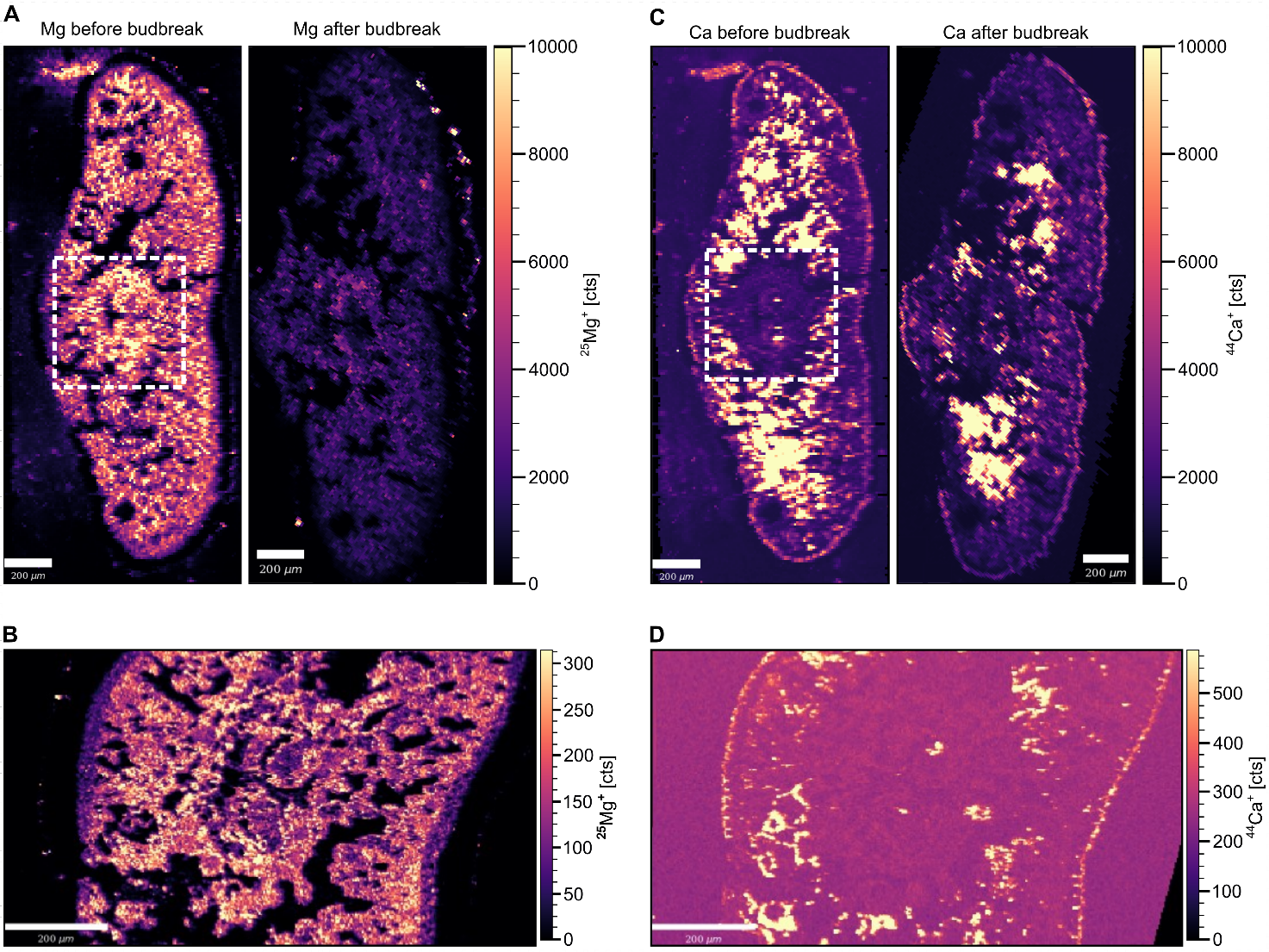
**

**Figure S3. Magnesium and Ca distribution in cryosections of one-year-old needles prior to and after budbreak.**

Tissue mapping of ^25^Mg and ^44^Ca was performed on the same cryosections prepared from one-year-old needles using LA-ICP-MS. Signal is presented in counts per second (cps). (A) Heatmaps present ^25^Mg distribution in needles harvested before and after budbreak. (B) Close-up image of ^25^Mg in a vascular bundle (region marked with the dashed line in image A) before budbreak. (C) Heatmaps of the ^44^Ca distribution in needles harvested before and after budbreak. (D) ^44^Ca localization in a vascular region in a needle before budbreak (region marked with the dashed line in image D).

**
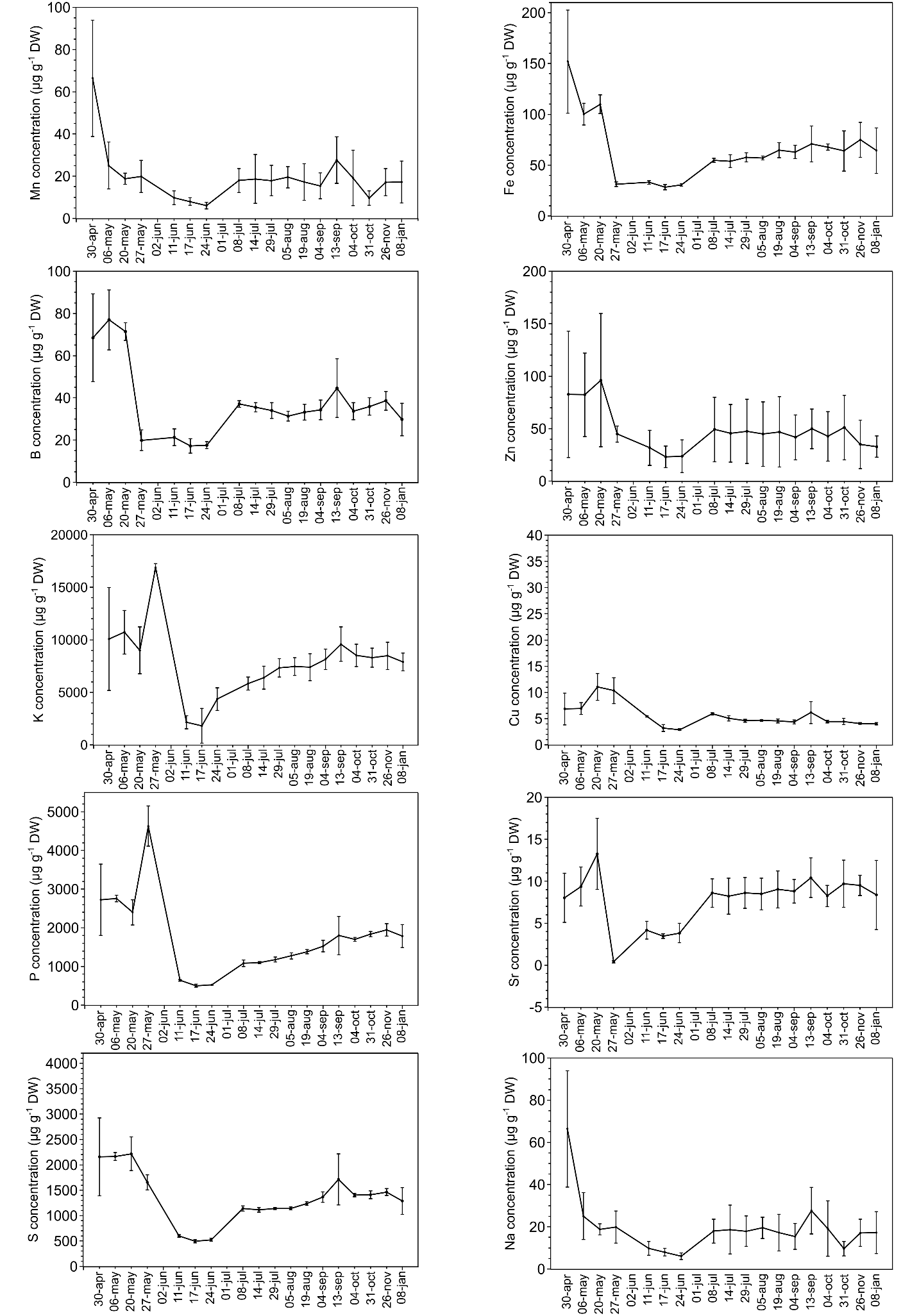
**

**Figure S4. Annual mineral fluxes in one-year old needles of Nordmann fir.**

Concentrations of elements were measured in one-year-old needles using ICP-OES. Charts present mean concentration values (±SD, *n* ≤ 3).


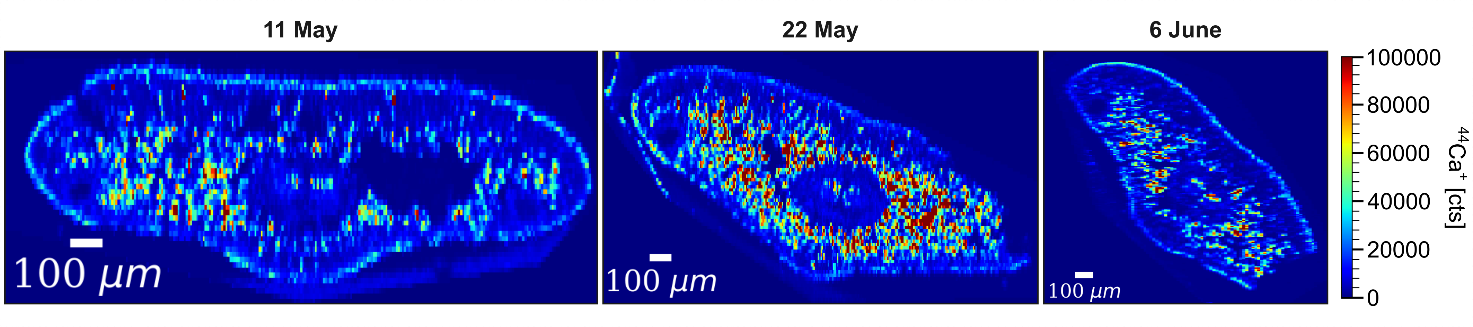


**Figure S5. Ca distribution in one-year-old needles before, during and after budbreak.**

Tissue mapping of ^44^Ca using LA-ICP-MS. Heatmaps present ^44^Ca distribution in cross-sections of resin-embedded one-year-old needles (at three harvest times, presented in counts per second (cps)).


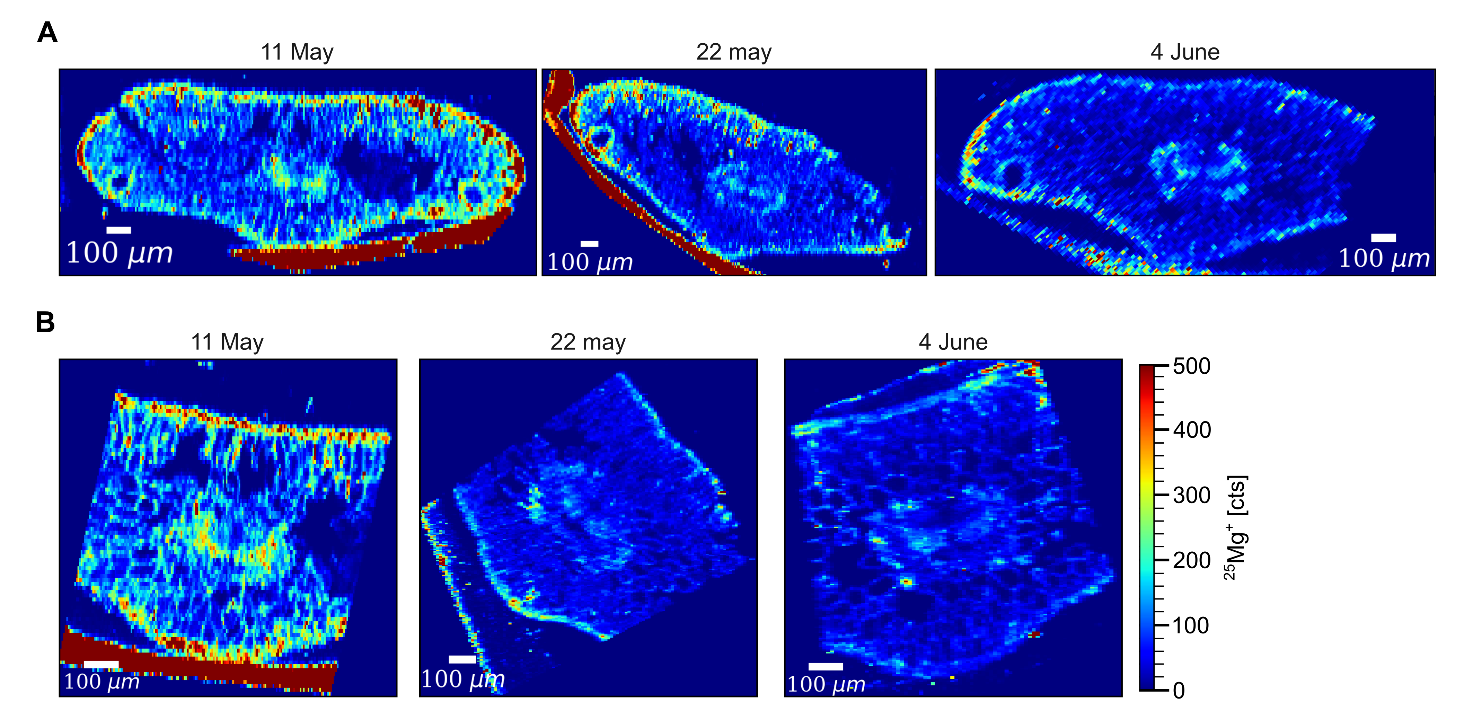


**Figure S6. Magnesium distribution in one-year-old needles before and after budbreak.**

Tissue mapping of ^25^Mg using LA-ICP-MS presented in counts per second (cps). Images show ^25^Mg distribution in cross-sections of resin-embedded whole one-year-old needles (A) and close-up images of vascular bundle region (B) in samples harvested at three timepoints.


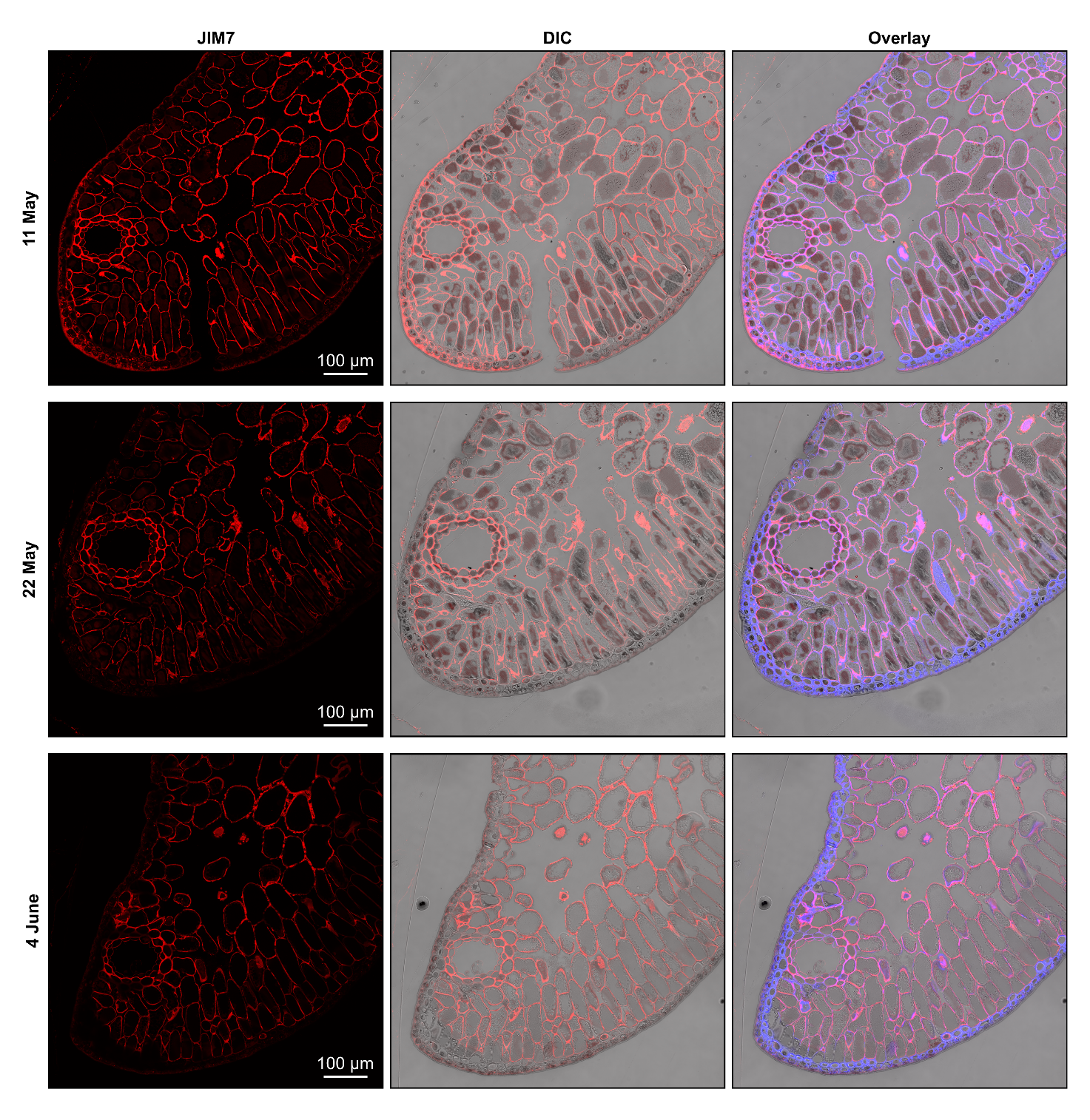


**Figure S7. Pectin distribution in the mesophyll of one-year-old needles before and after budbreak.**

Homogalacturonan with a high degree of methylesterification detected using a specific monoclonal antibody (JIM7, left panels) in resin-embedded sections harvested at three timepoints. The differential interference contrast images overlapped with the JIM7 signals (DIC, middle panels) and overlay images of JIM7, DIC and Calcofluor White (blue colour) (Overlay, right panels).

**Table S1. Annual Ca fluxes in current and one-year old needles of Nordmann fir.**

Concentrations of Ca were measured in current and one-year-old needles using ICP-OES. Presented are values for individual trees.

|  | **One-year-old needles** | | | **Current year needles** | | |
| --- | --- | --- | --- | --- | --- | --- |
|  | **Tree 1** | **Tree 2** | **Tree 3** | **Tree 1** | **Tree 2** | **Tree 3** |
| **30-apr** | 17024,61 | 9326,98 | 11043,6 |  |  |  |
| **06-maj** | 15206,65 | 14921,04 | 12217,45 |  |  |  |
| **20-maj** | 22734,44 | 19356,98 | 13890,86 |  |  |  |
| **27-maj** | 880,55 | 693,55 | 687 | 5692,79 | 20996,8 | 533,82 |
| **02-jun** | - | - | - | 924,44 | 656,66 | - |
| **11-jun** | 6029,13 | 7122,42 | 5367,01 | 1415,55 | 1183,96 | - |
| **17-jun** | 4953,58 | 5395,22 | 5560,56 | 1332,15 | 1627,23 | 1256,79 |
| **24-jun** | 6051,65 | 6127,21 | 4315,7 | 1314 | 2759,8 | 1851,99 |
| **01-jul** | - | - | - | 3493,3 | 2744,78 | - |
| **08-jul** | 12629,74 | 12097,52 | 10768,34 | 3436,97 | 3339,22 | - |
| **14-jul** | 12167,94 | 12477,4 | 9299,97 | 6481,38 | 3558,05 | 2405,76 |
| **29-jul** | 13121,23 | 12914,67 | 10713,84 | 5032,63 | 4774,2 | 3438,68 |
| **05-aug** | 13010,32 | 12263,28 | 10350,84 | 4954,06 | 4774,98 | 3484,98 |
| **19-aug** | 13929,1 | 13204,89 | 10494,65 | 5824,37 | 5501,76 | 3774,38 |
| **04-sep** | 11739,87 | 13496,6 | 12390,93 | 5516,19 | 5886,32 | 3925,22 |
| **13-sep** | 11981,8 | 14473,71 | 20239,27 | 7358,25 | 6972,62 | - |
| **04-okt** | 11400,39 | 12744,8 | 11593,41 | 5518,99 | 5109,91 | 4167,63 |
| **31-okt** | 13025,84 | 15588,61 | 10701,23 | 2740,6 | 5150,24 | 4188,78 |
| **26-nov** | 12325,48 | 14089,21 | 13141,52 | 5692,34 | 5342,06 | - |
| **08-jan** | 9829,54 | 16232,04 | 3952,68 | 4065,88 | 6548,39 | 12801,1 |

-, missing value.
